# Association of Lumbar Sagittal Curvature Profiles with Musculoskeletal Disorders: A Pilot Radiographic Study

**DOI:** 10.3390/diagnostics16091291

**Published:** 2026-04-25

**Authors:** Yu-Li Wang, Shu-Wei Huang, Hsuan-Yu Chen, Kuei-Chen Lee, Chao-Min Cheng

**Affiliations:** 1 Department of Surgery, Hualien Armed Forces General Hospital, Hualien 971, Taiwan; yuli0329@yahoo.com.tw; 2 International Intercollegiate Ph.D. Program, National Tsing Hua University, Hsinchu 300, Taiwan; 3 Department of Applied Science, National Taitung University, Taitung 950, Taiwan; judyya1022@gmail.com; 4 Department of Radiology, Tri-Service General Hospital, National Defense Medical University, Taipei 114, Taiwan; penguin0916@livemail.tw; 5 Department of Physical Medicine and Rehabilitation, Tri-Service General Hospital, National Defense Medical University, Taipei 114, Taiwan; 6 Institute of Biomedical Engineering, National Tsing Hua University, Hsinchu 300, Taiwan

**Keywords:** lumbar sagittal alignment, lumbar lordosis, diagnostic radiography, curvature profiling, musculoskeletal disorders

## Abstract

**Background/Objectives:** Altered lumbar sagittal alignment is associated with degenerative and mechanical musculoskeletal disorders. However, conventional angle-based measurements, such as the Cobb angle, may not fully reflect the overall curvature pattern of the lumbar spine. This pilot study investigated whether curvature distribution profiling on sagittal lumbar radiographs is associated with clinically confirmed lumbar musculoskeletal disorders. **Methods:** This retrospective pilot study included 50 adults who underwent standing sagittal lumbar radiography. Patients were classified as disease-positive or disease-negative according to radiographic findings, short-term clinical follow-up, and chart review. Vertebral body centroids from T12 to S1 were manually identified to construct continuous lumbar curvature profiles. Curvature height was normalized to a standardized baseline for cross-case comparison. Distribution patterns of curvature deviation were analyzed between groups. Total curvature was also calculated as a quantitative descriptor of overall lumbar bending, and group-wise comparisons were performed using the Kruskal–Wallis test. **Results:** The disease-negative group showed a predominantly unimodal and symmetric curvature distribution, whereas the disease-positive group showed greater dispersion at both hypo-lordotic and hyper-lordotic extremes. The hypo-lordosis subgroup demonstrated a more consistent deviation pattern, whereas the hyper-lordosis subgroup partially overlapped with the disease-negative distribution. These pattern-based findings suggest that deviation from a central curvature profile, rather than lordosis magnitude alone, may be associated with lumbar musculoskeletal disorders. In exploratory quantitative analysis, total curvature showed a distributional shift in the disease-positive group, although the overall between-group difference did not reach statistical significance (*p* = 0.096). **Conclusions:** Curvature distribution profiling may provide complementary morphological information in conjunction with conventional angle-based assessment. Both reduced and exaggerated lumbar curvature patterns were observed in association with lumbar musculoskeletal disorders. Larger studies are needed to validate these preliminary findings and to determine the clinical relevance of this approach.

## 1. Introduction

Low back pain is one of the most common complaints in clinical practice and affects individuals across a wide range of age groups, occupations, and activity levels [1,2,3,4]. Its multifactorial nature has highlighted the need for comprehensive assessment strategies that integrate structural evaluation with functional and interventional perspectives [5]. Globally, low back pain accounts for a substantial number of outpatient visits and contributes significantly to disability-adjusted life years (DALYs) [6,7,8,9], imposing a considerable burden on healthcare systems. Although its etiology is diverse, mechanical and degenerative disorders of the lumbar spine remain among the most common underlying causes [10,11,12]. In epidemiological terms, low back pain remains a major public health problem across regions. Globally, it was estimated to affect 619 million people in 2020, with projections rising to 843 million by 2050. In the United States, the prevalence of chronic low back pain increased from 3.9% in 1992 to 10.2% in 2006, whereas in Europe the 1-month prevalence has been reported to be 44.6%, ranging from 33.4% to 67.7% across countries. These data further support the view that low back pain is a heterogeneous condition and that its radiographic manifestations may extend beyond deformity-based or single-angle assessment alone [13,14,15]. These pathological processes may be reflected on radiographs through changes in alignment, segmental relationship, or overall sagittal morphology, even when they are not fully captured by a single angular parameter.

Plain radiographs remain a widely used first-line imaging tool for evaluating orthopedic conditions such as spondylosis, spondylolisthesis, and spinal deformity [16,17,18]. Although sagittal lumbar radiographs have limited ability to assess soft tissue abnormalities, including disc herniation, they continue to play an important role in evaluating spinal alignment and curvature, particularly in primary care and resource-limited settings [19,20,21,22].

**Figure 1:**
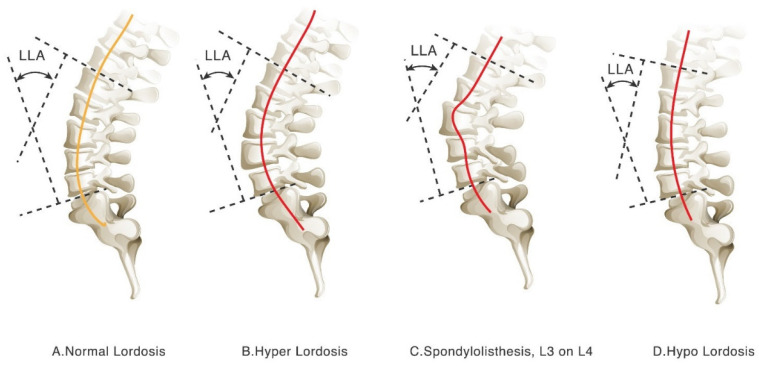
Limitations of Cobb-based lumbar lordosis assessment. Figure 1 illustrates four representative lumbar sagittal alignment patterns: (**A**) normal lordosis, (**B**) hyper-lordosis, (**C**) spondylolisthesis at L3–L4, and (**D**) hypo-lordosis. Cases with similar Cobb angles may still demonstrate substantially different overall curvature configurations. In particular, Cobb-based measurements may have limited ability to distinguish normal curvature from pathologic sagittal alignment or to adequately characterize relatively straight lumbar profiles. In contrast, curvature-based visualization provides a more intuitive representation of global lumbar alignment differences. The orange solid line represents the normal lumbar curvature pattern, whereas the red solid lines represent disease-associated lumbar curvature patterns, including hyper-lordosis, hypo-lordosis, or abnormal vertebral alignment due to spondylolisthesis.

Among radiographic measurements, the Cobb angle has long been regarded as the standard method for quantifying spinal curvature. Originally developed for scoliosis assessment [23,24,25], it has also been widely applied to the evaluation of lumbar lordosis and sagittal curvature [26,27,28,29]. Many studies have used Cobb angle-based parameters, such as the lumbar lordosis angle (LLA), to investigate associations between sagittal profile and clinical symptoms [30,31,32]. However, this approach has recognized limitations, including reduced reproducibility, sensitivity to vertebral level selection, and limited ability to characterize the overall complexity of lumbar curvature. Recent studies have also questioned the adequacy of sagittal Cobb angle measurements for evaluating lumbar lordosis [33,34,35,36]. Because Cobb angle measurements are derived from only two selected vertebral landmarks, they may not fully represent the multi-segmental morphology of the lumbar lordotic curve [37,38,39,40]. Discrepancies between the LLA and the lumbar lordotic curve (LLC) further suggest that manual measurement and observer variation may influence interpretation (Figure 1).

To address these limitations, several alternative radiographic indices have been proposed, including pelvic incidence–lumbar lordosis mismatch (PI-LL), sagittal vertical axis (SVA), vertex level measurements, lumbosacral angle (LSA), lumbosacral joint angle (LSJA), and tangential radiologic assessment of the lumbar lordosis angle (TRALLA) [41,42,43,44]. However, many of these parameters still rely primarily on angular descriptions or require full-spine imaging and detailed anatomical reference points, which may limit their routine use in standard lumbar radiograph interpretation [45,46,47]. As interest in personalized spine assessment continues to grow, additional curvature-based approaches are needed to better capture morphological variation in sagittal lumbar alignment.

Recent advances in image processing and geometric analysis have created new opportunities for curvature quantification [48,49]. Rather than relying solely on endpoint-based angular measurements, these approaches aim to describe the overall morphology of the lumbar spine and may provide more refined information for characterizing sagittal imbalance or degenerative change.

In this pilot study, we applied a curvature distribution profiling method to sagittal lumbar radiographs to visualize lumbar curvature patterns and to examine their association with clinically confirmed lumbar musculoskeletal disorders. By focusing on overall curvature morphology, this study aimed to determine whether curvature-based profiling could provide complementary radiographic information alongside conventional angle-based measures such as the Cobb angle [50]. We hypothesized that patients with clinically confirmed lumbar musculoskeletal disorders would show greater deviation from a central lumbar curvature profile than disease-negative individuals on standing sagittal lumbar radiographs. The originality of the present study lies not in curvature mathematics itself, but in the use of normalized cross-case curve-distribution profiling to evaluate deviation patterns on routine sagittal lumbar radiographs. This study is important because it explores whether routine sagittal lumbar radiographs can provide an additional pattern-based descriptor of lumbar disease in conjunction with conventional angle-based assessment. Rather than replacing advanced imaging, the present approach was intended to examine whether conventional radiography can be used more effectively as an early structural assessment tool.

Such an approach may be particularly useful in routine radiographic interpretation, where rapid visual assessment of overall curvature pattern may provide information not readily captured by discrete angular measurements. Unlike conventional lordosis indices that summarize alignment using selected angular relationships, the present method was designed to depict the overall curvature profile directly from vertebral alignment.

## 2. Materials and Methods

### 2.1. Patient Selection and Data Sources

This retrospective pilot study was conducted using standing sagittal lumbar radiographs obtained from the Department of Surgery, Armed Forces Hualien General Hospital, Taiwan. A total of 50 radiographs from outpatients who underwent lumbar spine examination between 1 December and 30 December 2023 were retrospectively collected. The study protocol was approved by the Institutional Review Board of Tri-Service General Hospital (IRB No. C202405032). Because only anonymized imaging data were used, the requirement for informed consent was waived.

### 2.2. Inclusion and Exclusion Criteria

The inclusion criteria were as follows:

(1) Age ≥ 18 years;
(2) Availability of a complete and clear standing sagittal lumbar radiograph including T12 to S1;
(3) No lumbar radiographic examination within the preceding 6 months, in order to reduce potential confounding from recent interventions or acute spinal events.

The exclusion criteria were as follows:

(1) History of spinal surgery;
(2) Presence of congenital spinal anomalies (e.g., hemivertebra or spina bifida);
(3) History of spinal trauma with fracture;
(4) Incomplete imaging data or poor image quality.

### 2.3. Group Classification

All cases were independently reviewed by an orthopedic surgeon and a radiologist. Group classification was based on the combination of initial radiographic findings, clinical records, and imaging or clinical follow-up within 3 months after the index examination.

#### 2.3.1. Disease-Negative Group

Patients without radiographic evidence of lumbar degenerative pathology at the index examination who showed no new lumbar symptoms during follow-up and required no additional spine-related imaging or intervention within 3 months.

#### 2.3.2. Disease-Positive Group

Patients with clinically confirmed degenerative lumbar disorders, such as disc herniation, spondylolisthesis, or facet joint degeneration, based on radiographic findings, chart review, and subsequent imaging evaluation (MRI or CT) or clinical intervention within 3 months [51,52,53].

This classification was not based solely on whether additional imaging was performed, but on the combination of radiographic findings, short-term clinical follow-up, and chart-confirmed clinical diagnosis, in order to reduce potential misclassification bias. The disease-positive group intentionally included clinically heterogeneous lumbar disorders because the aim of this pilot study was not to isolate disease-specific biomechanical mechanisms, but to examine whether different lumbar pathologies might share detectable deviation patterns in overall sagittal curvature. Given that lumbar disorders often arise from multiple interacting structural and soft-tissue factors, the present exploratory grouping was intended to capture the resultant geometric alteration rather than the isolated effect of a single disease entity. Disagreements between the two readers were resolved by consensus discussion. However, formal inter-observer agreement statistics were not calculated, and the consistency of manual landmark selection was therefore not quantified in this pilot study.

### 2.4. Image Processing and Curve Construction

For each case, the centroid of each vertebral body from T12 to S1 was manually identified, and these points were sequentially connected to construct the lumbar lordotic curve (LLC). A straight baseline was defined by connecting the centroids of T12 and S1, and its horizontal length was normalized to a scale of 0 to 100. The vertical distance from each vertebral centroid to this baseline was then measured to characterize the curvature profile. This normalization procedure was used to reduce the influence of individual differences in spinal size and curve length and to allow standardized comparison across cases.

**Figure 2:**
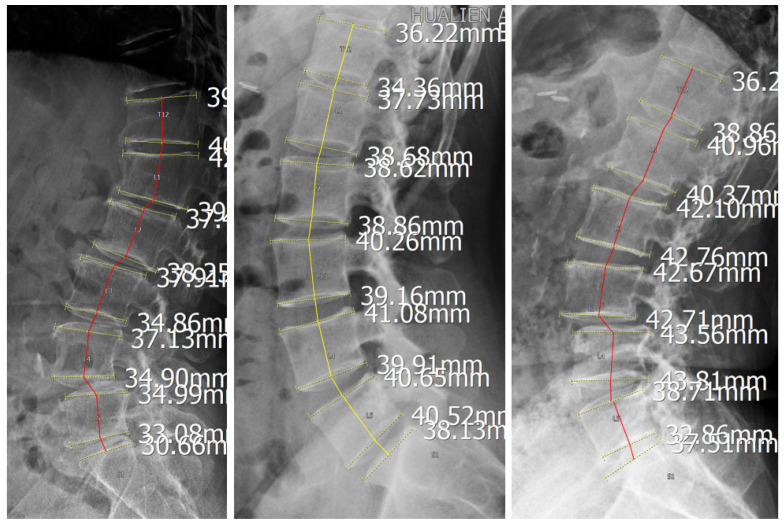
**Construction of the lumbar lordotic curve on sagittal radiographs.**  Figure 2 shows the construction of the lumbar lordotic curve on a sagittal lumbar radiograph by sequentially connecting the centroids of the vertebral bodies from T12 to S1.

**Figure 3:**
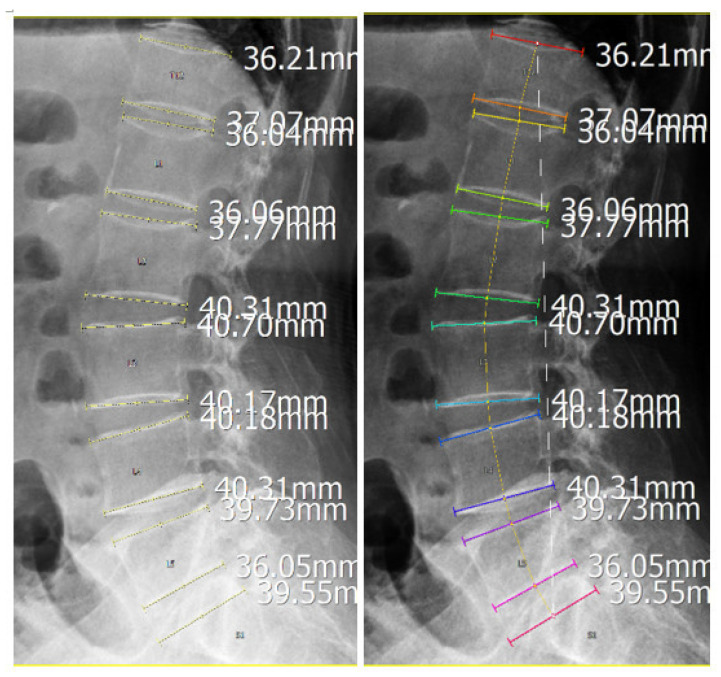
**Normalization and alignment of lumbar curvature profiles.** Colored solid lines indicate vertebral endplates identified by the imaging system, whereas the colored dashed line represents the curve connecting vertebral midpoints used to depict lumbar alignment. The white dashed line indicates the baseline connecting the inferior endplate of T12 and superior endplate of S1 used for normalization. Labels L1–L5 and S1 are vertebral identifiers generated by the imaging system.

For group-based visual assessment, each curve was colour-coded according to disease status, with yellow lines representing disease-negative cases and red lines representing disease-positive cases (Figure 2). A median curvature line was calculated for each group to represent its characteristic sagittal profile (Figure 3). Group comparisons focused on qualitative differences in curvature distribution and deviation from the central profile.

All image processing and plotting were performed using custom in-house tools. Landmark identification, curve construction, and profile visualization were conducted manually without automated segmentation or AI-assisted analysis.

### 2.5. Curvature Quantification and Statistical Analysis

In addition to visual curve profiling, curvature-based quantification was performed to further characterize the geometric properties of the lumbar lordotic curve. Curvature (*κ*) was defined using the parametric form of the curve, as follows:

κ =|x^′ y^″− y^′ x^″ |/ (x^′2+ y^′2)^(3/2)

Here, *x* and *y* represent the coordinates of the fitted lumbar curve, and primes denote the first- and second-order derivatives with respect to the curve parameter. Total curvature was then calculated as the integral of curvature along the arc length of the curve:

Total curvature=∫κ(s)ds

This parameter was used as a quantitative summary of overall lumbar geometric bending. Group-wise comparisons of total curvature among the three curvature pattern groups were performed using the Kruskal–Wallis test. Because this was a pilot study with a limited sample size, the statistical analysis was considered exploratory and was used to support, rather than define, the observed imaging patterns. Accordingly, subgroup findings were interpreted descriptively and were not intended to support definitive inferential conclusions.

## 3. Results

**Figure 4:**
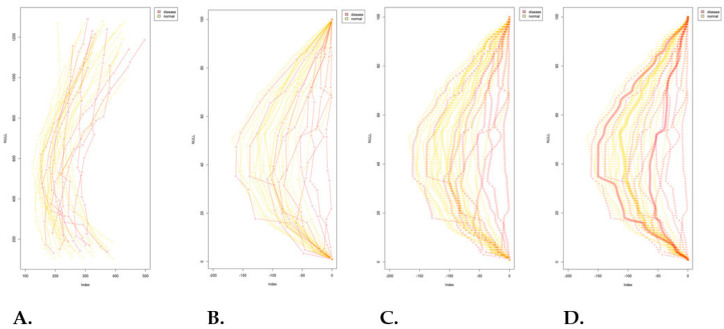
**Distribution patterns of normalized lumbar curvature profiles.** (**A**) Lumbar curvature profiles from all included cases were collected for analysis. (**B**) After normalization of curve size and alignment of the starting position, a central reference curvature profile was obtained. (**C**) The disease-negative group (yellow curves) showed a predominantly unimodal and symmetric distribution, whereas the disease-positive group (red curves) demonstrated broader dispersion toward both extremes of the distribution. (**D**) Subgroup analysis of the disease-positive cases showed two distinct patterns: a hyper-lordosis subgroup with partial overlap with the disease-negative distribution, and a hypo-lordosis subgroup with a shifted and comparatively narrower distribution.

The distribution of the study cohort by group, sex, and age is summarized in Table 1. The final dataset included 50 cases, comprising 32 disease-negative and 18 disease-positive cases. Within the disease-positive group, 11 cases were categorized as hyper-lordosis and 7 as hypo-lordosis. Fifty lumbar sagittal curvature patterns were analyzed (Figure 4A). After normalization, superimposed curves formed a central reference profile (Figure 4B). The disease-negative group (yellow curves) showed a predominantly unimodal and symmetric distribution, consistent with a typical lumbar curvature pattern. In contrast, the disease-positive group deviated from the reference profile and extended toward both extremes of the distribution, suggesting a bimodal tendency (Figure 4C,D).

**Figure 5:**
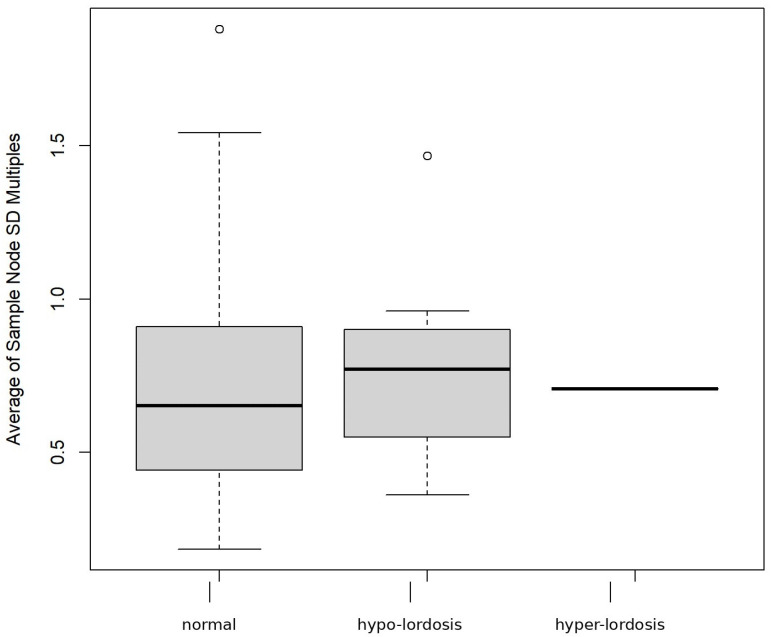
**Variability of lumbar curvature profiles across study groups.** Figure 5 illustrates the distribution of lumbar curvature profiles across the study groups, including the normal group and the two disease-associated subgroups, namely hypo-lordosis and hyper-lordosis. In the boxplot, the central line represents the median, the box represents the interquartile range, the whiskers indicate the data range, and circles indicate outlying observations. Boxplot analysis revealed a broader distribution in the normal group, whereas both disease-associated subgroups showed relatively reduced variability. The hyper-lordosis subgroup partially overlapped with the normal group, while the hypo-lordosis subgroup demonstrated a more distinct displacement in curvature.

Further stratification of the disease-positive group revealed two distinct sub-patterns: one characterized by reduced lumbar lordosis (hypo-lordosis subgroup in Figure 5) and the other by exaggerated lumbar curvature (hyper-lordosis subgroup in Figure 5). The hyper-lordosis subgroup overlapped considerably with the disease-negative group, indicating that increased curvature alone did not clearly distinguish disease status in this cohort. By contrast, the hypo-lordosis subgroup showed a narrower distribution and a lower median profile, indicating a more uniform geometric pattern among affected cases (Figure 4 and Figure 5).

The disease-negative group displayed a broader curvature range, with a wider interquartile span and a relatively higher median profile. By comparison, the hyper-lordosis subgroup showed minimal interquartile variation and a concentrated median profile, which may reflect either limited case numbers or relative structural homogeneity within this subgroup (Figure 5).

**Figure 6:**
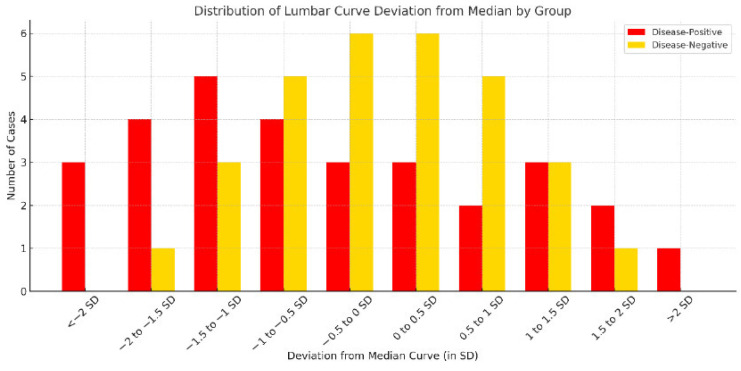
**Histogram of lumbar curvature deviation by disease status.** Figure 6 presents a histogram of lumbar curvature deviation using standard-deviation-based intervals. Compared with the disease-negative group, the disease-positive group showed a broader distribution across more extreme deviation categories, particularly beyond ±1.5 SD, indicating greater dispersion of curvature patterns in affected cases.

To further illustrate curvature deviation across groups, Figure 6 presents a histogram of lumbar curvature distribution according to distance from the group median (±1 SD, ±1.5 SD, and ±2 SD). The disease-negative group was primarily concentrated within ±1 SD of the median, consistent with a relatively stable central curvature pattern. In contrast, the disease-positive group showed a wider distribution, with more cases located beyond ±1.5 SD in both directions. This finding indicates that disease-positive cases tended to cluster at the extremes of curvature deviation.

To further quantify these geometric differences, total curvature was calculated from the continuous lumbar curve model for each subject. Kruskal–Wallis testing showed that the overall difference among the three groups did not reach statistical significance (*p* = 0.096). However, density distribution analysis demonstrated a visible shift in the disease-positive group relative to the disease-negative group. These results suggest that total curvature may provide a preliminary quantitative descriptor of geometric variation, but they do not establish statistically significant group separation in the present pilot cohort.

## 4. Discussion

To our knowledge, this pilot study is among the first to apply curvature distribution profiling to characterize lumbar disorders based on sagittal radiographic deviation patterns. The disease-negative group showed a relatively centralized and symmetric curvature profile, whereas the disease-positive group demonstrated broader deviation toward both extremes of lumbar curvature. These findings are consistent with previous reports suggesting that abnormal lumbar lordosis patterns, whether reduced or exaggerated, may be associated with lumbar disorders [33,48,49]. Our results therefore suggest that chronic lumbar disease may involve deviation in lumbar curvature in either direction rather than a simple increase or decrease in a single angular parameter. The disease-positive group was intentionally defined as a clinically heterogeneous category to examine whether different lumbar disorders might share a common tendency toward deviation from the central curvature profile. Accordingly, the findings should be interpreted as exploratory pattern-based observations rather than disease-specific biomechanical conclusions, particularly given the limited subgroup sizes in this pilot cohort.

The present findings are also in line with previous studies indicating that curvature deviation may be more informative than lordosis magnitude alone when evaluating lumbar disease [28,29,30,31,32,33,48,49,50]. In the present study, the distribution of larger lumbar curvature values in the disease-positive group partially overlapped with that of the disease-negative group, suggesting that exaggerated lordosis alone may not sufficiently distinguish diseased from non-diseased cases. In contrast, reduced lumbar curvature was more concentrated in the disease-positive group, supporting the potential relevance of hypo-lordotic morphology in chronic low back pain. This pattern may be associated with a straighter lumbar alignment and possible compensatory thoracolumbar adaptation; however, the underlying mechanism and any relationship to functional instability could not be determined in the present study. Recent studies have also reported altered postural control and reduced muscular endurance in patients with lumbar spondylosis, supporting the concept that morphological deviation may reflect underlying functional imbalance [54]. Conventional measures such as the Cobb angle may not fully capture these geometric complexities [28,29,33,39,49]. Although direct numerical comparison with established radiographic parameters such as the Cobb angle, PI-LL, and SVA was not performed in the present study, future work should evaluate the diagnostic value and additional contribution of curvature profiling in conjunction with these standard indices.

Low back pain remains a clinically important condition, and improving the radiographic assessment of lumbar disorders remains relevant to routine practice [55]. Conventional angle-based methods have been widely applied to the evaluation of lumbar lordosis and spondylolisthesis, but they may provide only limited representation of global lumbar morphology and can be influenced by manual measurement variability. Although several alternative radiographic approaches have been proposed [36,37,39,40,41,42], evidence remains limited regarding the relationship between overall sagittal curvature patterns and low back pain beyond segment-based angular comparisons [23,49,56,57].

Unlike prior approaches that primarily focused on individual curvature measurement or alternative geometric fitting, the present framework emphasizes normalized cross-case profile comparison and deviation-pattern interpretation. Whereas conventional indices such as the Cobb angle and related lordosis measures provide discrete angular estimates based on selected landmarks, curvature profiling was designed to describe the overall geometric configuration of the lumbar spine more directly. In practice, this visual profiling framework may offer a straightforward way to recognize atypical curvature patterns on standard lumbar radiographs before more advanced imaging or quantitative assessment is considered. In this context, sagittal curvature modeling may assist routine radiographic interpretation by providing a whole-curve view of lumbar morphology, particularly when conventional angle-based measures appear inconclusive or do not fully reflect the overall sagittal configuration. Clinically, this may serve as a practical adjunct to standard radiographic assessment rather than a replacement for existing measurements. Previous studies have examined LLA as a potential predictive tool for low back pain [1,44,45,46,58], but lumbar instability, which is considered an important contributor to chronic low back pain, has not been fully explained by angle-based parameters alone [19,25,54,59,60]. Abnormal lumbar curvature has been recognized as a potential risk factor [61,62,63,64], and in older adults, chronic low back pain is often associated with reduced lumbar lordosis [31,65,66]. Although thoracolumbar compensation and ligamentous or degenerative changes have been proposed as possible explanations [65,67,68], sufficiently integrated methods for assessing overall lumbar curvature remain limited. Larger studies are needed to validate these observations and to determine whether curvature-based profiling has practical value in radiographic assessment. The present approach is intended as a descriptive adjunct to conventional radiographic assessment and should not be interpreted as demonstrating superiority over established radiographic measurements.

## 5. Limitations

This study has several limitations related to its pilot design and methodological framework. First, the analysis was based exclusively on static sagittal lumbar radiographs and therefore did not directly assess dynamic spinal instability. Although this study was motivated by the broader clinical need to identify early biomechanical changes associated with lumbar disorders, the proposed curve-profiling approach was intended to characterize static sagittal lumbar morphology. The observed distribution patterns should therefore be interpreted as preliminary morphological indicators rather than direct evidence of dynamic dysfunction. Further validation using motion-based imaging modalities such as flexion–extension radiographs or dynamic MRI is warranted.

Second, inter-observer agreement was not quantified using formal reliability metrics such as Cohen’s kappa or the intraclass correlation coefficient (ICC). Although all image interpretations were independently performed by a board-certified radiologist and an orthopedic specialist, and discrepancies were resolved by consensus, the absence of quantified reliability measures remains a methodological limitation. Because this was an exploratory pilot study primarily intended to assess the feasibility of curvature distribution analysis, formal inter-rater testing was not performed. Future studies should incorporate inter-rater reliability analysis to improve reproducibility. Because the vertebral centroids were manually identified, the lack of formal intra-observer and inter-observer repeatability data remains an important methodological limitation, and formal reproducibility testing will be essential in future validation studies. In this pilot study, manual centroid selection was used to establish the feasibility of the curve-profiling framework under controlled visual assessment and to ensure anatomically consistent landmark placement across cases. Future studies may incorporate automated or AI-assisted vertebral landmark detection to improve efficiency, standardization, and reproducibility, particularly in larger datasets and routine clinical workflows.

Third, the sample size was limited to 50 cases, which restricted statistical power and generalizability. In particular, the overlap observed between disease-positive and disease-negative cases in the hyper-lordosis subgroup could not be fully interpreted because of the limited number of subjects. In addition, although a preliminary curvature-derived quantitative analysis was performed, the study remained underpowered for definitive inference. These issues should be addressed in future studies with larger cohorts. In addition, because the disease-positive group included heterogeneous lumbar disorders with potentially different biomechanical relationships to sagittal curvature, the present grouping also limited disease-specific interpretation of the observed curvature patterns. Moreover, demographic characteristics, detailed pathology categories, and non-radiographic comorbidities were not systematically collected in the original study design. Therefore, the present study could not evaluate the potential influence of these factors on pain presentation or curvature patterns. Accordingly, subgroup analyses based on age, sex, BMI, pathology type, or comorbidity status were not feasible in the present study.

Finally, no direct numerical comparison was performed between the proposed method and established radiographic parameters such as the Cobb angle, PI-LL, or SVA. Therefore, the additional contribution of curvature distribution profiling relative to standard measurements could not be determined in the present study. Although the present approach offers an alternative way to describe sagittal alignment, its diagnostic performance, including sensitivity, specificity, and predictive value, remains to be established. Future studies should include direct comparative analyses with established radiographic parameters to clarify the additional value of curvature distribution profiling in clinical practice.

## 6. Conclusions

This pilot study showed that lumbar curvature distribution profiling can depict sagittal morphological variation associated with lumbar musculoskeletal disorders on standing radiographs. Both reduced and exaggerated lumbar curvature patterns were observed in disease-positive cases, suggesting that deviation from a central curvature profile may provide complementary morphological information alongside conventional angle-based assessment. Although the present findings remain preliminary, curvature profiling may offer a complementary radiographic approach for describing sagittal lumbar morphology. Larger studies are needed to determine whether the observed visual and quantitative patterns remain reproducible in broader cohorts.

## Figures and Tables

**Table 1:** Distribution of the study cohort and lumbar curvature subgroups.

Group	*n*	Male, *n* (%)	Female, *n* (%)	Age, Mean ± SD
Total	50	26 (52%)	24 (48%)	57.3 ± 16.8
Disease-Negative	32	13 (40.6%)	19 (59.4%)	50.0 ± 14.8
Disease-Positive	18	13 (72.2%)	5 (27.8%)	70.3 ± 18.1
Hyper-lordosis	11	6 (54.5%)	5 (45.5%)	72.5 ± 6.8
Hypo-lordosis	7	7 (100%)	0 (0%)	66.9 ± 17.1

Abbreviation: SD, standard deviation. Note: Disease-positive cases were further categorized into hyper-lordosis and hypo-lordosis subgroups according to curvature pattern classification. Age is presented as mean ± SD, and sex is presented as *n* (%).

## Data Availability

The datasets used and/or analyzed during the current study are available from the corresponding author on reasonable request, subject to institutional and ethical regulations regarding de-identified clinical imaging data.
